# Proactive enrollment of parents to tobacco quitlines in pediatric practices is associated with greater quitline use: a cross-sectional study

**DOI:** 10.1186/s12889-016-3147-1

**Published:** 2016-06-24

**Authors:** Jeremy E. Drehmer, Bethany Hipple, Emara Nabi-Burza, Deborah J. Ossip, Yuchiao Chang, Nancy A. Rigotti, Jonathan P. Winickoff

**Affiliations:** Center for Child and Adolescent Health Research and Policy, Division of General Academic Pediatrics, Massachusetts General Hospital, 125 Nashua Street, Suite 860, Boston, MA 02114 USA; Tobacco Research and Treatment Center, Massachusetts General Hospital, Boston, MA USA; Department of Public Health Sciences, University of Rochester Medical Center, Rochester, NY USA; Harvard Medical School, Boston, MA USA

**Keywords:** Smoking, Tobacco, Pediatrics, Family practice, Parent, Smoking cessation, Secondhand smoke, Tobacco control, Quitline, Telephone counseling

## Abstract

**Background:**

Every U.S. state has a free telephone quitline that tobacco users can access to receive cessation assistance, yet referral rates for parents in the pediatric setting remain low. This study evaluates, within pediatric offices, the impact of proactive enrollment of parents to quitlines compared to provider suggestion to use the quitline and identifies other factors associated with parental quitline use.

**Methods:**

As part of a cluster randomized controlled trial (Clinical Effort Against Secondhand Smoke Exposure), research assistants completed post-visit exit interviews with parents in 20 practices in 16 states. Parents’ quitline use was assessed at a 12-month follow-up interview. A multivariable analysis was conducted for quitline use at 12 months using a logistic regression model with generalized estimating equations to account for provider clustering. Self-reported cessation rates were also compared among quitline users based on the type of referral they received at their child’s doctor’s office.

**Results:**

Of the 1980 parents enrolled in the study, 1355 (68 %) completed a 12-month telephone interview and of those 139 (10 %) reported talking with a quitline (15 % intervention versus 6 % control; *p* < .0001). Parents who were Hispanic (aOR 2.12 (1.22, 3.70)), black (aOR 1.57 (1.14, 2.16)), planned to quit smoking in the next 30 days (aOR 2.32 (1.47, 3.64)), and had attended an intervention practice (aOR 2.37 (1.31, 4.29)) were more likely to have talked with a quitline. Parents who only received a suggestion from a healthcare provider to use the quitline (aOR 0.45 (0.23, 0.90)) and those who were not enrolled and did not receive a suggestion (aOR 0.33 (0.17, 0.64)) were less likely to talk with a quitline than those who were enrolled in the quitline during the baseline visit. Self-reported cessation rates among quitline users were similar regardless of being proactively enrolled (19 %), receiving only a suggestion (25 %), or receiving neither a suggestion nor an enrollment (17 %) during a visit (*p* = 0.47).

**Conclusions:**

These results highlight the enhanced clinical effectiveness of not just recommending the quitline to parents but also offering them enrollment in the quitline at the time of their child's visit to the pediatric office.

**Trial registration:**

ClinicalTrials.gov, Identifier: NCT00664261

## Background

The United States Department of Health and Human Services has identified smoking cessation as a national health priority by setting objectives within Healthy People 2020 to reduce tobacco use prevalence and increase the use of smoking cessation services [1]. The 2014 report of the U.S. Surgeon General on smoking estimates that 480,000 people die annually from smoking-attributable diseases in the United States [2], and there is no safe level of exposure to tobacco smoke [3]. Children are disproportionately impacted by their parents’ tobacco use [4–6] yet parents who smoke often have limited access to healthcare services for themselves [7]. Parental smokers are frequently seen at the pediatric office [7, 8], and as a result, the pediatric office setting represents an ideal opportunity to connect parents with evidence-based smoking cessation services to help them become tobacco-free.

The Clinical Effort Against Secondhand Smoke Exposure (CEASE) intervention has been developed for the pediatric office setting to help parents quit smoking and reduce children’s exposure to tobacco smoke [9, 10]. Through the CEASE intervention, clinical and administrative staffs at pediatric offices were trained to work as a team to routinely and effectively address family tobacco use and exposure through identifying smokers and offering brief assistance with smoking cessation. This assistance comes through prescribing nicotine replacement therapy (NRT) and referring parents who smoke to free smoking cessation counseling services, including telephone quitlines [10, 11].

Quitlines are free state-specific telephone-based tobacco cessation services that help tobacco users quit through providing a variety of services, including one-on-one telephone counseling, cessation medications, and self-help materials. Smokers can receive quitline services by calling the quitline directly, through a fax referral from a healthcare provider’s office, or enrolling online in certain states. To complete a fax referral, a simple one-page fax referral form is completed by the smoker, signed by a healthcare provider (in some cases), and faxed to the quitline. Trained smoking cessation counselors from the quitline will then call the smoker at the telephone number indicated on the fax referral form. The smoker and the trained smoking cessation counselor formulate a smoking cessation plan and carry out the plan over a series of weeks; state quitlines offer a number of free counseling sessions to each smoker (varies by state), as well as additional educational materials. Medications, commonly nicotine replacement gum or patch, are sometimes provided for free to low-income and underinsured populations; distribution of free medication, however, is not always available and often depends on the funding from each state’s health department. Beyond the time it takes to hand out and fax in the form, there is no direct cost to healthcare providers for referrals.

Quitlines have a broad reach [12]. They can reduce barriers for access to treatment when compared to traditional healthcare services, and quitlines are accessible to populations commonly underserved by other programs [13]. Quitlines are a well-established evidence-based treatment [4, 12, 14–17], yet, despite the free cost of referrals, rates of quitline referrals for parents in the pediatric setting remain low [11]. In a previous paper that compared intervention practices trained in CEASE to usual care control practices, we showed that of the 981 smoking parents who visited pediatric practices in the control group at baseline, 0 % reported being enrolled at the visit in a tobacco quitline [11]. This is an unfortunate missed opportunity to help families become smoke-free as most parents report they would accept a quitline enrollment if it were offered to them from their child’s doctor [18].

Referrals to quitlines in the healthcare setting can be delivered through instructing smokers to call the quitline on their own or in a proactive manner such as sending a fax referral from a healthcare provider’s office. Previous research conducted in pediatric healthcare settings suggests that the proactive model where parents complete a quitline fax referral form at the healthcare visit yields greater quitline use [7] compared to instructing parents to call the quitline on their own which had resulted in few calls to the quitline [19]. A study conducted in Australia shows provider-initiated proactive quitline enrollments using fax referral forms led to a quitline utilization rate of 74 % among adult patients recruited from a preoperative clinic [20]. Likewise, a study of adult primary care practices in Oregon demonstrated that proactive qutiline enrollment to the Oregon Tobacco Quit Line using fax referral forms led to a substantial increase in quitline use. While 59 % of fax-referred smokers were successfully contacted proactively by the Oregon Tobacco Quit Line, 41 % of fax-referred smokers were not successfully connected by telephone and thus did not receive smoking cessation counseling from the quitline [21]. The large percentage of smokers that are referred by fax and do not end up connecting with the quitline is often cited as a drawback of the proactive referral method because quitlines then devote substantial resources to make unsuccessful call-back attempts to large numbers of smokers [22]. A similar trend was found with fax referrals of smokers seen in Mississippi dental offices where smokers were more likely to quit if they connected with the quitline, though only a small number of people who received the fax referral ultimately connected and received counseling from the quitline [23]. While repeated call attempts due to unanswered calls or disconnected phone numbers is a significant burden for quitlines [22], other promotional strategies such as mass media campaigns designed to drive smokers to quitlines may be more expensive [21]. Additionally, the population of smokers referred by fax at a healthcare provider office have generally different characteristics than smokers who respond to quitline mass media campaigns [24]. Thus, many individuals who are referred to quitlines in the healthcare setting may not be the same individuals most likely to respond to promotional advertisements for quitlines.

To better understand quitline usage by parents who were seen in the pediatric outpatient setting, we examined whether proactive enrollment (compared to none or suggestion only) was associated with greater quitline use, after controlling for other factors that could influence the likelihood of using a quitline. Proactive enrollment involves asking the parent who smokes to complete a fax referral form at the time of the visit, which is then faxed to the quitline by the practice staff. The quitline then attempts to contact the parent directly upon receiving the fax referral. Pediatric providers may be ambivalent about enrolling parents to the quitline because they are uncertain how often their referrals will actually be effective in connecting the parents to the quitline [13]. Pediatric healthcare providers will benefit from knowing to what extent proactive enrollments to the quitline are effective and if they are more or less likely to result in use of the quitline compared to a provider suggestion to call the quitline.

We also examined a question posed by Anderson & Zhu [13] in their detailed review of tobacco quitlines which asked whether quitlines are as effective at generating cessation outcomes for people who are proactively enrolled by healthcare providers compared to people who take the initiative to contact quitlines on their own. This is an important question because provider-referred quitline users in other contexts have been observed to utilize the services less and are, on average, less ready to quit compared to self-referred clients [24, 25]. In contrast, an Australian cluster randomized trial conducted with general practitioners demonstrated an increase in smoking cessation rates for patients at 3-months if their practitioner was encouraged to send a fax a referral form to a telephone quitline compared to seeing a practitioner instructed to provide standard of care for smokers [17]. If smoking cessation rates of those who are proactively enrolled by a pediatric healthcare provider are even somewhat similar to those who call quitlines on their own then it would suggest that integrating quitline enrollment into the routine pediatric office workflow could be effective at helping a large number of parents quit smoking. In this paper we determined the rate of quitline use in addition to the cessation rate of parents based on the type of referral received from their child’s healthcare provider to evaluate the overall impact of proactive enrollment of parents to the quitline compared to provider suggestion to use the quitline.

## Methods

The data used in this analysis were collected as part of a cluster randomized controlled trial of the Clinical Effort Against Secondhand Smoke Exposure (CEASE) intervention. The CEASE intervention is a simple method for pediatric offices to systematically address parental smoking and reduce tobacco smoke exposure in families [11]. Twenty pediatric practices from 16 states participated and practices were randomized to control and intervention arms of the study. Healthcare providers at intervention practices were trained to deliver the CEASE intervention, which included training to proactively enroll smoking parents to the quitline by asking parents to complete a fax referral form while at the visit and faxing it to the quitline. The practices were recruited from the Pediatric Research in Office Settings network, the practice-based research network of the American Academy of Pediatrics (AAP).

### Participant enrollment

Research assistants attempted to complete post-visit exit interviews with all parents exiting the pediatric office. Parents were eligible to be enrolled in the study if they: indicated that they had smoked a cigarette, even a puff, in the last 7 days, were the parents or legal guardians of the child seen at the visit, were 18 years or older, were English speaking, had a working telephone number, and agreed to sign an informed consent form to enroll in the study. Parents who enrolled in the study at the baseline exit interview were called at 12 months to complete a follow-up telephone survey.

### Measures & statistical analysis

Quitline use, assessed at the 12-month telephone follow-up interview, was defined in this study as having talked about smoking with someone at a quitline. A multivariable analysis using a logistic regression model was conducted to examine which factors measured at baseline were associated with quitline use at 12 months. Variables included in the model were planning to quit in the next 30 days, planning to quit in the next 6 months, frequency of smoking (daily versus non-daily), not receiving any type of referral to use the quitline (none) versus being proactively enrolled at the baseline visit, receiving only a suggestion to use the quitline versus being proactively enrolled at the baseline visit, age, gender, education, race, and practice study arm assignment. The receipt of a quitline referral was assessed by parent self-report at the post-visit exit interview and the practice study arm assignment variable was assigned based on whether or not the practitioners at the practice where the parent was seen had received training in the CEASE intervention.

Parents were asked during the post-visit exit interview and at the 12-month telephone follow-up interview if he or she received a suggestion from anyone, at that visit or at any visit since enrolling in the study respectively, to use the telephone quitline to help quit smoking. If the parent reported at the post-visit exit interview or the 12-month telephone follow-up interview that he or she received a suggestion, the parent was then asked if he or she was enrolled in the quitline to help quit smoking at that visit or at any visit since enrolling in the study respectively. An example of a suggestion to use the quitline is providing the parent with the telephone number for the quitline and leaving it up to the parent to call on his or her own after the visit. The providers participating in the CEASE intervention arm of the study were encouraged to proactively enroll smoking parents to the quitline using the fax referral form during the office visit, though parents who smoked were not randomly assigned to receive a particular type of quitline referral. Rates of quitline use at the 12-month follow-up were compared using a chi-square test for tobacco users who were proactively enrolled at the pediatric office to those who only received a suggestion from the provider to use the quitline to those who received neither during the baseline office visit or at a subsequent visit within the 12-month follow-up period. We also compared self-reported cessation rates using a chi-square test among parents who talked with someone at a quitline who were proactively enrolled in the quitline, who only received a suggestion to use a quitline, and who were not referred by either method to a quitline during the baseline visit or within the 12-month follow-up period. Self-reported smoking cessation at the time of the 12-month interview was defined as not smoking a cigarette, even a puff, within the past 7 days. All analyses were conducted by using generalized estimating equation techniques to take into account provider clustering. SAS version 9.3 (SAS Institute, Cary, NC) was used for all analyses.

## Results

Of the 1980 parents enrolled, 1355 (68 %) completed a 12-month telephone interview and of those 139 (10 %) reported talking with a quitline about smoking (15 % intervention versus 6 % control; *p* < .0001). Demographic and baseline characteristics of the parents who completed the 12-month telephone interview are presented in Table 1. The follow-up rates of parents enrolled at baseline who completed the 12-month telephone interview were not significantly different for those who received a proactive enrollment to the quitline (65.9 %), received only a suggestion to use the quitline (70.6 %) or received neither (68.3 %) at the baseline visit (*p* = 0.71). The results of the multivariable logistic regression model are presented in Table 2. Parents who were Hispanic (aOR 2.12 (1.22, 3.70)), black (aOR 1.57 (1.14, 2.16)), planned to quit smoking in the next 30 days (aOR 2.32 (1.47, 3.64)), and had attended an intervention practice (aOR 2.37 (1.31, 4.29)) were more likely to have talked with a quitline. Parents who only received a suggestion from a healthcare provider to use the quitline (aOR 0.45 (0.23, 0.90)) and those who were not proactively enrolled and did not receive a suggestion from a healthcare provider (aOR 0.33 (0.17, 0.64)) were less likely to talk with a quitline than those who were proactively enrolled in the quitline at the baseline visit.

**Table 1:** Characteristics of enrolled parents (*N* = 1980)

Demographic/baseline characteristic	Parents enrolled in study at baseline post-visit exit interview (*N* = 1980)^a^	Parents interviewed at 12-months (*n* = 1355)^a^	Talked with a quitline about smoking at 12-months (*n* = 139)^a^
		n (%)	n (%)
Parent Age			
<30	1058	696 (65.8)	64 (9.2)
≥30	921	658 (71.4)	75 (11.4)
Race and ethnicity			
Hispanic	216	143 (66.2)	22 (15.4)
Non-Hispanic >1 race	55	38 (69.1)	3 (7.9)
Non-Hispanic black or African-American	307	193 (62.9)	22 (11.4)
Non-Hispanic Asian	9	6 (66.7)	2 (33.3)
Non-Hispanic Native American or other	47	32 (68.1)	3 (9.4)
Non-Hispanic white	1331	931 (69.9)	84 (9.0)
Sex			
Male	429	256 (59.7)	29 (11.3)
Female	1549	1097 (70.8)	110 (10.0)
Education			
≤ High School	1222	800 (65.5)	81 (10.1)
> High School	751	550 (73.2)	58 (10.5)
Frequency of Smoking			
Daily	1679	1157 (68.9)	125 (10.8)
Non-daily	300	198 (66.0)	14 (7.1)
Planning to quit			
Within 30 days	841	586 (69.7)	84 (14.3)
Within 6 months	564	392 (69.5)	31 (7.9)
Not planning to quit	510	340 (66.7)	19 (5.6)
Study Arm			
Intervention	999	645 (64.6)	96 (14.9)
Control	981	710 (72.4)	43 (6.1)

^a^Parents with missing data were not included

**Table 2:** Adjusted odds ratios and 95 % confidence intervals for talking with a quitline at 12-months (*N* = 1355)

Baseline characteristic	Adjusted OR (95 % CI)
Parent Age (≥30 vs. < 30)	1.21 (0.77, 1.90)
Race (Hispanic vs. White)	2.12 (1.22, 3.70)
Race (Black vs. White)	1.57 (1.14, 2.16)
Race (Other vs. White)	1.38 (0.62, 3.08)
Sex (Male vs. Female)	1.08 (0.68, 1.73)
Education (> High School vs. ≤ High School)	1.07 (0.73, 1.56)
Frequency of Smoking (Daily vs. Non‐daily)	1.65 (0.87, 3.12)
Plan to Quit in 30 days	2.32 (1.47, 3.64)
Plan to Quit in 6 months	1.25 (0.75, 2.07)
Quitline Referral Type (Suggestion only vs. Proactively enrolled at visit)	0.45 (0.23, 0.90)
Quitline Referral Type (None vs. Proactively enrolled at visit)	0.33 (0.17, 0.64)
Study Arm (Intervention vs. Control)	2.37 (1.31, 4.29)

Parents who were proactively enrolled in a quitline during one of their child's visits were more likely than those who received a suggestion only to report using the quitline at follow-up. Specifically, 48 out of 254 parents (19 %) who received a suggestion at the baseline or at a subsequent visit to use the quitline but were never proactively enrolled in the quitline reported talking with a quitline about smoking during the 12 month follow-up period whereas 37 out of 82 parents (45 %) who were proactively enrolled in the quitline at the baseline visit or at a subsequent visit reported talking with a quitline about smoking during the 12 month follow-up period. Only 54 out of 1019 (5 %) parents who did not receive a suggestion to use quitline and were not proactively enrolled in the quitline at the baseline visit or at a subsequent visit ended up talking with a quitline. Results are presented in Table 3.

**Table 3:** Quitline use by type of quitline referral at baseline visit or a subsequent visit (*N* = 1355)

	Total (*N* = 1355)	Talked with a quitline about smoking at 12-months
	*N*	*n* (%)
Did not receive a suggestion to use quitline and not enrolled for quitline at a visit	1019	54 (5.3)
Only received suggestion to use quitline at a visit	254	48 (18.9)
Proactively enrolled in the quitline at a visit	82	37 (45.1)

(chi-square (2) = 42.7, *p* < 0.0001)

Self-reported smoking cessation rates were not significantly different among parents who talked with a quitline about smoking regardless of receiving a proactive enrollment to the quitline (19 %), receiving only a suggestion to use the quitline (25 %) or receiving neither (17 %) at the baseline visit or during a subsequent visit within the 12-month follow-up period (chi-square (2) = 1.52, *p* = 0.47). Results are presented in Table 4.

**Table 4:** Self-reported cessation at 12-months by referral type among parents who talked with a quitline (*N* = 139)

	Total (*N* = 139)	Quit smoking	Quit smoking
	*N*	*n*	% (95 % CI)
Did not receive a suggestion to use quitline and not enrolled for quitline at a visit	54	9	16.7 % (8.2 %–28.4 %)
Only received suggestion to use quitline at a visit	48	12	25.0 % (15.1 %–36.9 %)
Proactively enrolled in the quitline at a visit	37	7	18.9 % (8.9 %–32.4 %)

(chi-square (2) = 1.52, *p* = 0.47)

## Discussion

This study examined quitline usage by parents seen in the pediatric outpatient setting to determine what type of referral was associated with greater quitline use. Pediatrician’s suggestion to use the tobacco quitline was associated with an increased likelihood of quitline use by parents, but proactive enrollment to the quitline at the time of the visit was associated with more than double the rate of quitline utilization compared to suggestion alone. As shown in Table 3, being proactively enrolled in the quitline at a visit, the highest level of intervention in this study, showed the strongest association with quitline use, suggesting that higher levels of intervention to connect smoking parents to quitlines may yield greater amounts of quitline users in pediatric practices. The findings presented in the current study are consistent with findings from the limited number of previous studies that have looked at the association between referral type and quitline utilization for parents within a pediatric setting [7, 19]. Proactive enrollment in the quitline in adult healthcare settings using fax referral forms also found significantly higher quitline utilization rates like we did in the current study [17, 20, 21, 26]. Therefore, the existing evidence suggests that changing clinic workflows to not only recommend quitlines to parental smokers, but also enroll parents to the quitline at the point of service should result in referrals that generate a higher yield of quitline users.

Quitline promotional efforts within pediatric healthcare settings will be furthered strengthened by knowing that proactive quitline enrollments were associated with a similar likelihood of smoking cessation among quitline users when compared to encouraging parents enroll in the quitline on their own [13]. A previous study conducted on Arizona Smokers Helpline callers found that a healthcare provider suggestion to use the quitline and a proactive quitline enrollment by fax were each significantly more likely to result in cessation than callers who were not referred in either way to the quitline by a provider, though the quit rates following a provider suggestion to use the quitline or proactive quitline enrollment were not statistically different from each other [27]. We did not find a statistically significant different rate of quitting among quitline users who did not receive a suggestion to use quitline and were not enrolled for quitline, only received suggestion to use quitline, or were proactively enrolled in the quitline.

Because our study demonstrated that proactive enrollment to the quitline greatly increases the rate of quitline use compared to provider suggestion alone, we would expect the number of quitters to increase in practices that use the proactive referral model compared to provider suggestion alone. At the time of our study, the participating practices were utilizing paper-based medical records. A pilot quitline eReferral system that was developed using Epic electronic health record software was tested in a project with the Wisconsin Tobacco Quit Line and showed that adult tobacco users visiting healthcare clinics were more likely to receive a referral to the quitline when utilizing the eReferral method compared to the traditional method of using a paper fax referral form [28]. Developing similar efficient electronic quitline referral systems that fit into the clinical workflow for smoking parents seen in the pediatric setting should help maximize the benefits demonstrated in this study [29]. Electronic referral systems that allow for enhanced communication between the pediatric healthcare provider and the quitline might be helpful to coordinate care and ensure smokers get successfully connected to the quitline [24].

The results of our cessation outcome analysis presented in Table 4 should be interpreted cautiously due to a small sample size. Additional limitations of this study include the lengthy 12-month follow-up period when a parent could have used a quitline thus increasing the potential for recall bias, the potential for a parent to have been influenced during the follow-up period to use a quitline outside of the context of their child’s medical visit, and the possibility that the self-reported cessation outcome was caused by factors other than quitline use that were not accounted for in this study. Parental motivation to quit may have influenced the type of quitline referral they received. For instance, a parent could have declined to complete a quitline fax referral form after it was offered to them by a healthcare provider. This study also relied on parent self-report data and the use of cross-sectional data, which precludes inference of causality.

We found that black and Hispanic parents were more likely than white parents to use the quitline. A previous study of California Smokers Helpline users had similar results and found that black smokers were also more likely than whites to use the service [30]. Reasons for these observed racial disparities among quitline users are not entirely known, though minority groups suffer disproportionate health and economic disparities from tobacco-related diseases when compared to whites [31]. Quitlines are also available to everyone regardless of income, insurance status, or availability of transportation, and the near universal availability of tobacco quitlines compared to other tobacco cessation services may be at least partly responsible for the increased use of quitlines among minorities who may have more barriers to accessing other forms of smoking cessation treatment. While the availability of quitlines has improved access to tobacco dependence treatment, a recent study showed significant disparities persist in the long-term cessation rates between high and low socioeconomic status individuals who use free proactive quitlines [32]. More intensive tobacco cessation service delivery to low socioeconomic status individuals may be needed to improve cessation rates. For instance, distribution of free or subsidized nicotine replacement therapy has been shown to increase quitline usage [33] and is associated with higher smoking cessation rates among individuals receiving telephone counseling [34, 35].

We also found that quitlines are more likely to be used by parents who are planning to quit within the next 30 days. This suggests that parents who are contemplating a quit attempt in the near future may be a particularly important group to target with quitline enrollment, as they were more likely to connect with the quitline and use the service. Some quitlines evaluate callers’ readiness to quit as a criteria for allocating more intensive counseling services [36], and it is unknown to what degree this practice may have influenced the results observed in our study. For instance, some quitlines screen smokers and provide counseling only to those people who are ready to quit [36] in an effort to maximize the overall impact on the population with the limited funding received by quitlines [37]. When possible, future research should take into account quitline eligibility protocols used to allocate counseling services when comparing cessation outcomes across different referral types.

## Conclusions

Our results highlight the importance of not just recommending the quitline to parents but also offering parents the opportunity to enroll in the quitline at the time of their child's visit to the pediatric office. By enrolling parents who smoke in the quitline, pediatric healthcare providers maximize the likelihood that underserved families use this free evidence-based program to help become tobacco-free.

## Abbreviations

AAP, American Academy of Pediatrics; CEASE, Clinical Effort Against Secondhand Smoke Exposure

## References

[1] U.S. Department of Health and Human Services. Healthy people 2020: Tobacco use. https://www.healthypeople.gov/2020/topics-objectives/topic/tobacco-use?topicid=41. Accessed 17 March 2016.

[2] US Department of Health and Human Services (2014). The Health Consequences of Smoking- 50 Years of Progress: A Report of the Surgeon General.

[3] US Department of Health and Human Services (2006). The Health Consequences of Involuntary Tobacco Smoke: A Report of the Surgeon General.

[4] Fiore MC, Jaen CR, Baker TB (2008). Treating tobacco use and dependence: Update. Clinical Practice Guideline.

[5] DiFranza JR, Lew RA (1996). Morbidity and Mortality in Children Associated With the Use of Tobacco Products by Other People. Pediatrics.

[6] Bailey SL, Ennett ST, Ringwalt CL (1993). Potential mediators, moderators, or independent effects in the relationship between parents’ former and current cigarette use and their children's cigarette use. Addict Behav.

[7] Winickoff JP, Buckley VJ, Palfrey JS, Perrin JM, Rigotti NA (2003). Intervention With Parental Smokers in an Outpatient Pediatric Clinic Using Counseling and Nicotine Replacement. Pediatrics.

[8] Newacheck PW, Stoddard JJ, Hughes DC, Pearl M (1998). Health insurance and access to primary care for children. N Engl J Med.

[9] Winickoff JP, Hipple B, Drehmer J, Nabi E, Hall N, Ossip DJ (2012). The Clinical Effort Against Secondhand Smoke Exposure (CEASE) intervention: A decade of lessons learned. J Clin Outcomes Manag.

[10] Winickoff JP, Nabi-Burza E, Chang Y, Regan S, Drehmer J, Finch S (2014). Sustainability of a parental tobacco control intervention in pediatric practice. Pediatrics.

[11] Winickoff JP, Nabi-Burza E, Chang Y, Finch S, Regan S, Wasserman R (2013). Implementation of a parental tobacco control intervention in pediatric practice. Pediatrics.

[12] Zhu SH, Anderson CM, Tedeschi GJ, Rosbrook B, Johnson CE, Byrd M (2002). Evidence of real-world effectiveness of a telephone quitline for smokers. N Engl J Med.

[13] Anderson CM, Zhu SH (2007). Tobacco quitlines: looking back and looking ahead. Tob Control..

[14] Lichtenstein E, Glasgow RE, Lando HA, Ossip-Klein DJ, Boles SM (1996). Telephone counseling for smoking cessation: rationales and meta-analytic review of evidence. Heal Educ Res.

[15] Stead LF, Lancaster T (2001). Telephone counselling for smoking cessation. Cochrane Database Syst Rev..

[16] Ossip-Klein DJ, McIntosh S (2003). Quitlines in North America: evidence base and applications. Am J Med Sci.

[17] Borland R, Balmford J, Bishop N, Segan C, Piterman L, McKay-Brown L (2008). In-practice management versus quitline referral for enhancing smoking cessation in general practice: a cluster randomized trial. Fam Pract. England.

[18] Winickoff JP, Tanski SE, McMillen RC, Hipple BJ, Friebely J, Healey EA (2006). A national survey of the acceptability of quitlines to help parents quit smoking. Pediatrics.

[19] Winickoff JP, Hillis VJ, Palfrey JS, Perrin JM, Rigotti NA (2003). A smoking cessation intervention for parents of children who are hospitalized for respiratory illness: the stop tobacco outreach program. Pediatrics.

[20] Wolfenden L, Wiggers J, Campbell E, Knight J, Kerridge R, Moore K (2008). Feasibility, acceptability, and cost of referring surgical patients for postdischarge cessation support from a quitline. Nicotine Tob Res.

[21] Bentz CJ, Bayley KB, Bonin KE, Fleming L, Hollis JF, McAfee T (2006). The feasibility of connecting physician offices to a state-level tobacco quit line. Am J Prev Med.

[22] Willett JG, Hood NE, Burns EK, Swetlick JL, Wilson SM, Lang DA (2009). Clinical Faxed Referrals to a Tobacco Quitline. Reach, Enrollment, and Participant Characteristics. Am J Prev Med.

[23] Gordon JS, Andrews JA, Crews KM, Payne TJ, Severson HH, Lichtenstein E (2010). Do faxed quitline referrals add value to dental office-based tobacco-use cessation interventions?. J Am Dent Assoc.

[24] Borland R, Segan CJ (2006). The potential of quitlines to increase smoking cessation. Drug Alcohol Rev.

[25] Song G, Landau AS, Gorin TJ, Keithly L (2014). Real-World Impact of Quitline Interventions for Provider-Referred Smokers. Am J Prev Med.

[26] Sheffer MA, Baker TB, Fraser DL, Adsit RT, McAfee TA, Fiore MC (2012). Fax referrals, academic detailing, and tobacco quitline use: a randomized trial. Am J Prev Med.

[27] Guy MC, Seltzer RGN, Cameron M, Pugmire J, Michael S, Leischow SJ (2012). Relationship between smokers’ modes of entry into quitlines and treatment outcomes. Am J Health Behav.

[28] Adsit RT, Fox BM, Tsiolis T, Ogland C, Simerson M, Vind LM, et al. Using the electronic health record to connect primary care patients to evidence-based telephonic tobacco quitline services: a closed-loop demonstration project. Transl Behav Med. 2014;1–9. 10.1007/s13142-014-0259-y PMC4167898 25264471

[29] Sharifi M, Adams WG, Winickoff JP, Guo J, Reid M, Boynton-Jarrett R (2014). Enhancing the Electronic Health Record to Increase Counseling and Quit-Line Referral for Parents Who Smoke. Acad Pediatr.

[30] Zhu SH, Gardiner P, Cummins S, Anderson C, Wong S, Cowling D (2011). Quitline utilization rates of African-American and white smokers: The California experience. Am J Heal Promot.

[31] U.S. Department of Health and Human Services. Tobacco Use Among U.S. Racial/Ethnic Minority Groups-African Americans, American Indians and Alaska Natives, Asian Americans and Pacific Islanders, and Hispanics (1998). A Report of the Surgeon General.

[32] Varghese M, Sheffer C, Stitzer M, Landes R, Brackman SL, Munn T (2014). Socioeconomic disparities in telephone-based treatment of tobacco dependence. Am J Public Health.

[33] Grigg M, Glasgow H (2003). Subsidised nicotine replacement therapy. Tob Control.

[34] Macleod ZR, Charles MA, Arnaldi VC, Adams IM (2003). Telephone counselling as an adjunct to nicotine patches in smoking cessation: a randomised controlled trial. Med J Aust.

[35] Stead LF, Perera R, Lancaster T (2007). A systematic review of interventions for smokers who contact quitlines. Tob Control..

[36] Cummins SE, Bailey L, Campbell S, Koon-Kirby C, Zhu SH (2007). Tobacco cessation quitlines in North America: a descriptive study. Tob Control..

[37] Perry RJ, Keller PA, Fraser D, Fiore MC (2005). Fax to quit: a model for delivery of tobacco cessation services to Wisconsin residents. WMJ.

